# Individual variation within parasite communities of endangered African lions

**DOI:** 10.1098/rsos.250501

**Published:** 2025-10-29

**Authors:** Nyeema C. Harris, Jane Hallam

**Affiliations:** ^1^ Applied Wildlife Wildlife Lab, School of the Environment, Yale University, New Haven, CT, USA

**Keywords:** carnivore, Felidea, helminths, microbe, national park, West Africa

## Abstract

Prey depletion and human–wildlife conflict threaten the critically endangered West African populations of lion (*Panthera leo leo*), which occupy less than 1.1% of their historic range in West Africa. These threats may alter behaviour through prey selection and affect exposure to parasites to compromise their health. We extracted DNA from faecal samples collected in four national parks in Benin, Burkina Faso and Senegal to characterize haemoparasites, nemabiome and microbiome. We used microsatellite markers to differentiate individuals and five primer sets to complete molecular analyses. From 20 individuals (12 males and 8 females), we found significant differences in the species richness and composition for all parasite groups across host populations and individual lions. We detected haemoparasites, including *Babesia* and *Sarcocystis* species, along with *Blechomonas*, a kinetoplastid, all of which raise potential health concerns. The nemabiome was dominated by *Ancylostoma* species (hookworms) with additional detections of lungworms from the genera *Oslerus* and *Troglostrongylus*. Significant interactions were found between population-level microbiome richness and both nemabiome and haemoparasite diversity. Our study provides the first effort to determine the parasite diversity among West African lion populations using non-invasive metabarcoding. Our findings highlight metabarcoding as a powerful tool to assess spatial variation in health and parasite diversity metrics for an endangered apex predator.

## 1. Introduction

The importance of individual identification in behavioural and ecological assessments is being increasingly recognized, beyond just demographic estimates of population size [1,2]. Such discernment is particularly relevant in disease ecology to identify the subsets of populations most vulnerable to infection or individuals principally responsible for maintaining or spreading diseases. While some species are uniquely identifiable due to natural markings (e.g. *Giraffa camelopardalis* and *Equus grevyi*) that facilitate the use of camera trapping for non-invasive monitoring [3], discrimination among individuals for many carnivores of conservation concern rely on genetic sampling collected from scat or hair surveys. Scat collection also facilitates additional insights about an individual’s ecology through diet analysis and assessments of health through parasitological investigations [4,5]. Ultimately, it is the individual variation within animal populations that governs the contributions of species to the environment and their role within the ecosystem [6,7].

Mounting evidence demonstrates the role of parasites, inducing heterogeneity within populations and niche differentiation among individuals [8,9]. Parasites, by definition, cause some physiological harm to their host through morbidity and mortality effects on individuals that scale to threaten the persistence of species [10,11]. Across natural systems, infected individuals have been shown to behave differently with altered activity patterns, movement characteristics and habitat use [12,13]. Infected individuals also exhibit compromised body condition, energy and haematological parameters that govern their ecology, including diet and habitat choices [14,15]. Parasites themselves, however, also represent important aspects of biodiversity within systems and can aid individuals through digestion, immune function, competitive exclusion, coevolution and protection against pollutants [16,17]. Thus, characterizing the variation of parasite communities within host individuals informs behavioural phenotypes, patterns of biodiversity and ecosystem function under changing environmental conditions.

For social species, individual phenotypes emergent from variation in parasite infections may be dampened due to increased encounter rates in their shared environments and behaviours; although, the extent of parasite differentiation among group members also depends on subgroup structure and individual traits [18,19]. Sex, age and social rank within clans of spotted hyenas (*Crocuta crocuta*) affected the infection and survival probability of canine distemper virus in the Serengeti [20]. For giraffes, individuals that periodically engaged in interactions outside their primary social group experienced increased helminth infections [21]. Environmentally transmitted helminths were more prevalent in individual chimpanzees (*Pan troglodytes schweinfurthii*) that spent more time in the same place with more individuals rather than constrained to their grooming networks [22]. Not surprisingly, positive effects of sociality in relation to parasite infection exist widely across mammals [23]. However, sympatry with other host species that serve as reservoirs is also sufficient to promote the spread and maintenance of parasites among species not living in groups. Geographic overlap strongly predicts parasite sharing among mammal taxa as demonstrated in ungulates [24], primates [25] and carnivores [26]. With increasing global environmental and land-use changes, wildlife communities and their affiliates are reshuffling in a manner that affects spatial biodiversity patterns and heightens vulnerability of endangered species.

As some of the most threatened and ecologically significant vertebrates, carnivores structure communities and their slow life history traits and wide ranges make them highly vulnerable to anthropogenic pressures [27,28]. Habitat loss and subsequent prey depletion have resulted in historic range contractions with incessant concerns of population extirpations for extant species [29,30]. Additionally, carnivores are hampered by parasites and among the top mammal orders implicated for disease spillover of zoonotic viruses [31,32]. Despite harbouring a diverse and abundant community of zoonotic parasites, carnivores may serve as sentinels within an ecosystem. In this role, carnivores simply mirror or reflect the parasite assemblage in an area due to their often generalist nature, trophic level and roaming behaviours, but not always act themselves as important reservoirs or superspreaders for the causative disease agent [33]. Studying how parasite communities vary among individual social carnivores and their interactions within their hosts can reveal the evolutionary processes that shape these relationships and help anticipate potential consequences emergent from global changes [34].

Africa’s largest apex predator, the lion (*Panthera leo leo*)*,* currently exists in a small fraction of their total historic range across the continent, with critically endangered populations lingering throughout West Africa [30,35]. Host density, abundance or rarity are key determinants of parasite heterogeneity among individuals [36,37]. Given the vulnerability and ecological significance of African lions [38], coupled with their unique group-living social structure, understanding the variation in parasite communities among individuals becomes crucial to their conservation and ecosystem function. African lions benefit from group living through food acquisition, territory defence, reproductive success and cub survival [39]. Concurrently, their group size and social structure dynamics induce variation in the contribution of individuals to the persistence of pathogens within a pride and transmission between prides [40,41]. As habitats dwindle and lions abut human interfaces, exposure to pathogens from domestic spillover will increase, further altering the composition and heterogeneity of parasites among individuals [42,43].

Here, we ask how do parasite communities vary at the individual and population level within West African lions using faecal metabarcoding. We highlight three distinct parasite groups: haemoparasites, nemabiome and microbiome to test hypotheses of individual and population-level variation. Haemoparasites are transmitted by vectors like ticks and can have pathogenic effects on the survival and reproduction of their immunocompromised or naive hosts [44,45]. Nemabiome, comprising parasitic nematodes, influences the nutrition, immunity and fitness of their host [15]. As trophically transmitted parasites, nematodes often reflect the diet and foraging behaviour of their hosts, making them an ideal marker for understanding ecological interactions in carnivores [24]. The microbiome plays key roles in nutrient absorption, digestion and immune regulation, which are linked to variations in diet, environment and individual health [46,47]. Throughout the manuscript, the microbiome data obtained represent a subset of the total microbiome and are characterized using 16S-based bacterial community profiling. We hypothesized that nemabiome diversity would show the greatest individual variation due to differences in prey consumption and trophic transmission. We also expected greater population-level similarity among haemoparasites compared with the microbiome and nemabiome, as haemoparasites are more sensitive to environmental conditions and the study populations occur in similar environments. We expected the Senegal population to comprise a narrower network and less diverse assemblage of parasites compared with Burkina Faso and Benin due to smaller population sizes. Finally, we expected a positive correlation between nemabiome and microbiome diversity, but do not expect such correlation between these parasite groups and haemoparasites. Studies such as ours, that integrate multiple scales of parasite structure help further elucidate drivers of community assembly and host susceptibility to potential disease exposure, yielding new insights for mitigation measures to protect vulnerable species.

## 2. Methods

### 2.1. Study site and sample collection

**Figure 1.**
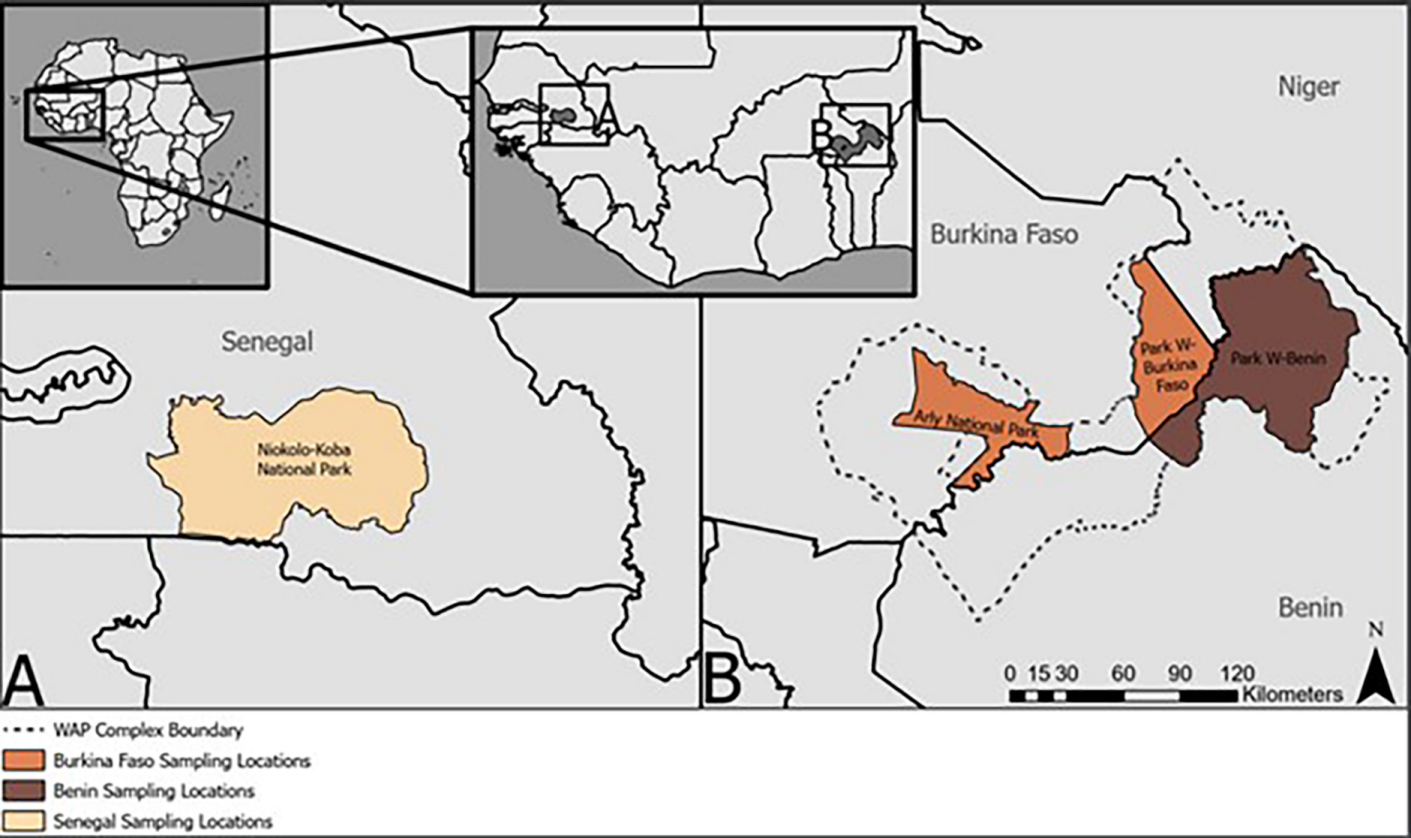
Study areas in West Africa of scat sample collection from critically endangered lion populations in protected areas in Senegal and the WAP complex of Burkina Faso and Benin.

We opportunistically collected carnivore faecal samples within the W-Arly-Pendjari (WAP) transboundary protected area of West Africa as well as from Senegal’s largest national park. Faecal samples nearly or completely desiccated were not collected. We collected subsamples, focusing on ends and interiors to minimize contamination with soil surface. Specifically, 36 deposits presumed to be lion were obtained from Park W-Benin, Park W-Burkina Faso and Arly National Park from 2016 to 2018 in the WAP complex, and from Niokola Koba National Park (NKNP) in Senegal in 2020 during the dry season (figure 1). While these protected areas are devoid of human settlements, villages do immediately surround the parks with communities accessing to obtain non-timber forest products and other resources (e.g. [48]). While the total lion population size is presently unknown for NKNP, recent estimates are 28−56 individuals within the core of the park [49]. In contrast, the estimated lion population for the WAP complex, though dated, was 246−466 individuals [35]. Over 90% of the critically endangered West Africa lion population resides in the WAP complex, a multi-use ecosystem shared with a myriad of human pressures and intensive livestock grazing [35,50]. Collected faecal samples from these four locales were preserved in ethanol, dimethyl sulfoxide saline solution buffer or RNAlater (Qiagen, USA), totalling 100 samples that represent replicates across faecal deposits. Samples were stored at −30°C until DNA extraction and extracted using the same protocol regardless of the previous storage method. We also obtained tissue and muscle from a lion cub resulting from an infanticide event to use as a positive control for molecular analyses.

### 2.2. DNA extraction and species identification

We employed an optimized version of the QIAamp Fast stool mini kit protocol (Qiagen, USA) for extraction of faecal samples using an average 244 mg of material. We checked for purity and concentration levels of DNA using a Nanodrop 2000 spectrophotometer (Thermo Fisher Scientific) and fluorometer (Qubit V2, Thermo Fisher Scientific), respectively. Before we began investigating parasite assemblages, we first confirmed samples were felids and then differentiated individuals using genotyping. We amplified DNA using the primers LIHYF (5′-ATGACCAACATTCGAAAATCWC-3′) and LIHYR (5′-ATGTGGGTSACTGATGAG-3′), which amplify a 206 bp fragment of the mitochondrial *cytochrome b gene* [51]. A blank control (reagents only) was included in all PCRs to monitor for contamination, along with a positive control of verified lion DNA. The subsequent PCR products were evaluated using electrophoresis on a 1.5% agarose gel with SYBR Safe gel stain (Thermo Fisher Scientific) and then Sanger sequenced. Forward and reverse sequences were aligned using Genious Prime v 10.1.2 (Biomatters Ltd.) software then blasted against the NCBI database to confirm their identity as African lion based on a minimum 98.6% identity. See electronic supplementary material for additional details on extraction protocol and PCR methods.

### 2.3. Microsatellite amplification for genotyping of individuals and molecular sex determination

Samples confirmed as lions were scored for allelic variability using nine polymorphic microsatellite primers (FCA077, FCA126, FCA031, FCA085, FCA096 and FCA045 [52], and Leo077, Leo045 and Leo281 [53]) to differentiate individuals and identify sample ‘recaptures’. Due to the potential of allelic drop-out, PCRs were conducted in triplicate and detections were only retained if they were consistent in at least two of three reactions (43% were retained). For each sample, the triplicate PCR amplicons were combined in equal volume (5 μl from each reaction, totaling 15  μl per sample) into a single pooled sample prior to purification and library preparation. This step was intended to minimize PCR stochasticity and ensure even representation across replicates. Following identification of individuals, sex was determined using X and Y chromosome-specific primers (SMCX17 and DBY7) [54]. PCRs were conducted in multiplex reactions, in triplicate per sample, to simultaneously amplify fragments of the *amelogenin* X gene and the Y-linked sex-determining region (*SRY*) gene. The *amelogenin* locus is expected to amplify in all samples containing sufficient nuclear DNA and thus serves as a positive PCR control, while the *SRY* locus should amplify only if a Y chromosome template is present and thus is used to assign sex. After amplification, PCR products were evaluated using 1.5% agarose gel with SYBR Safe DNA gel stain. Samples with one (XX) vs two (XY) bands were scored as females and males, respectively. Fragment analysis for genotyping and Sanger sequencing for host species ID were performed at the Keck DNA Sequencing Facility at Yale University.

### 2.4. Metabarcoding and bioinformatics

We used metabarcoding on the extracted DNA to characterize parasite diversity of haemoparasite, nemabiome and bacterial community. Four primer pairs were used to investigate parasite diversity (electronic supplementary material, table S1). Two groups of protozoan haemoparasites were targeted: the phylum Apicomplexa and the class Kinetoplastea. Studies have successfully detected blood parasites, such as malaria causing Plasmodium, from DNA extracted from faecal samples, despite challenges such as low target DNA and the presence of substances inhibitory to PCR amplification [55]. Again, PCR reactions were carried out in triplicate and pooled before sequencing. Optimal thermocycling conditions varied for PCRs across parasite groups (see electronic supplementary material for additional details). Of the 66 lion samples, 65 successfully amplified and underwent sequencing, along with the inclusion of an additional PCR negative control. Sequencing was carried out at the Yale Center for Genome Analysis (YCGA) using an Illumina NovaSeq with 2x200 bp chemistry. To target known haemoparasite diversity, we classified the Apicomplexa and Kinetoplastea amplicon sequence variants (ASVs) with the 18S reference dataset curated by the Ribosomal Database Project [56]. We applied taxonomic classifications for the nemabiome ASVs using a nematode-specific ITS2 rDNA database [57]. Finally, we derived the bacterial community ASVs using the Silva 138.1 database that was optimized for the classification of bacteria [58,59]. ASV identifications were made to species level, or otherwise, the lowest taxonomic rank. See electronic supplementary material for PCR settings and methods for discerning ASVs from the raw sequence as well as all sequence data and taxonomic assignments of parasite classifications.

### 2.5. Statistical analyses

Our aim was to investigate variation in the different parasite assemblages detected from faecal metabarcoding at individual and population levels. To visualize patterns of parasite composition among individuals, we calculated Bray–Curtis dissimilarities from the sample-by-species matrix and ordinated using non-metric multidimensional scaling (NMDS). We then used Analysis of similarities (ANOSIM) to determine significant differences between the detected community compositions among individuals, and similarity percentages (SIMPER) analysis to investigate the contributions of species to average dissimilarity within the *vegan* R package [60]. Hierarchical clustered heatmaps of the parasite assemblages were generated using the *pheatmap* package to investigate correlations and potential relationships [61]. Parasite co-occurrences were investigated using *cooccur* [62].

We determined species richness by summing the number of respective ASVs detected at both the sample level and aggregated to the individual lion level using the *vegan* package [60]. Differences in ASV richness were investigated using Levene’s test to assess the equality of variances in different samples, prior to repeated measures ANOVA. Given that each individual lion had more than one sample attributed to it, repeated measures ANOVA was used to account for the within-individual correlation across multiple samples. The effect size (here the generalized eta squared for repeated measures) was calculated using the *effectsize* package [63]. An analysis of variance was conducted and showed there was no statistically significant difference in ASV richness among the different preservation methods used for the faecal samples (Welch one-way ANOVA: *F*_(3.7, 3)_ = 5.78, *p* = 0.09). We investigated the relationships between haemoparasite, nemabiome and microbiome ASV richness using Spearman’s correlation coefficient, and with generalized linear models (GLMs) fitted with Poisson distribution and negative binomial distribution using the *MASS* package [64]. All analyses were completed in the R program (version 4.2.0).

## 3. Results

We collected scat samples from Park W-Benin, Park W-Burkina Faso, Arly National Park and Niokola Koba National Park and confirmed 24 deposits (comprised of 65 samples given replicates across preservation type) collected as *Panthera leo*. We were able to successfully genotype to identify 20 unique individuals (Benin = 2, Burkina Faso = 13 and Senegal = 5; table 1). One ambiguous individual shared alleles with two other individuals but did not amplify across all nine microsatellite sites, so could not be further distinguished. Of the 21 putative individuals, 8 females and 12 males were identified.

**Table 1.** ASV richness detections for each individual lion in the study across parasite groups collected from faecal samples in West Africa populations.

individual	sex	country	apicomplexan	kinetoplastid	microbiome	nemabiome
16.A	M	Burkina Faso	1	99	50	113
16.B	F		0	2	59	68
16.C	M		61	67	130	31
16.D	F		63	2	121	67
16.E	M		18	75	82	58
16.F	F		0	30	41	64
17.A	M		0	71	76	76
17.D	M		45	95	139	190
17.E	M		59	81	116	204
17.F	F		6	0	63	74
18.A	M		42	144	68	97
18.B	F		0	9	55	26
18.C	na		1	10	142	117
18.D	M		31	9	51	138
17.B	M	Benin	87	72	113	278
17.C	M		0	3	107	50
20.A	F	Senegal	7	3	66	59
20.B	M		49	67	57	156
20.C	F		13	323	104	67
20.D	F		75	0	44	86
20.E	M		59	120	138	292

### 3.1. Haemoparasite diversity

**Figure 2.**
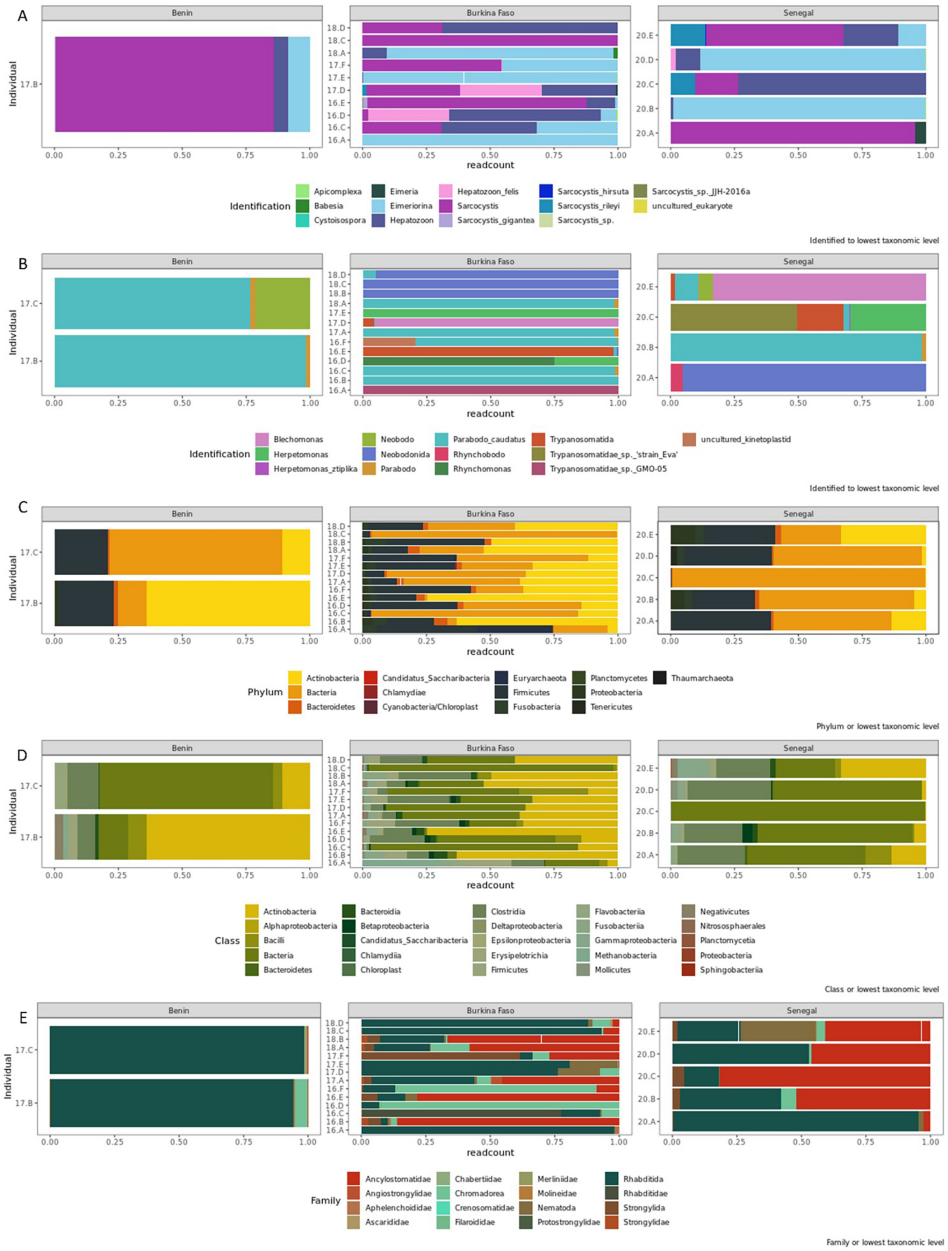
Stacked bar charts illustrating the diversity and proportion of haemoparasites in (A) apicomplexan, (B) kinetoplastid, (C) microbiome phyla and (D) microbiome classes, and (E) nemabiome family detections per individual lion and grouped by country.

We identified 148 apicomplexan ASVs from 16/21 individuals with richness per individual ranging from 1 to 87 where present (individual 17.B, a male from Benin; table 1). Mean ASV richness was 38.6 and varied by population (Benin: 43.5, Burkina Faso: 23.4, Senegal: 40.6). There was a near-significant effect of population on apicomplexan richness (*F*_(2, 13)_ = 3.244, *p* = 0.072), with an effect size of *η*² = 0.18. No significant difference in apicomplexan ASV richness was found between the sexes. Overall, apicomplexan ASVs comprised detections from the families Adeliorina, Conoidasida, Eimeriorina and Piroplasmorida (figure 2). The family Adeleorina was dominated by the genus *Hepatozoon* and included the species-level detection of *Hepatozoon felis* in 7 lion individuals. Within the Eimeriorina, sequences from the genus *Sarcocystis* were most abundant (14 lion individuals) and included detections up to species level of *Sarcocystis gigantea*, *S. hirsute* and *S. rileyi*. Additionally, there were two detections of *Eimeria* sp. (individuals 17.D and 20.A) and one of *Babesia* sp. (individual 18.A). *Sarcocystis* made up the majority of detections from lion samples collected in Benin (85.7%) while *Eimeria* dominated detections for lion samples from Burkina Faso and Senegal (73.8% and 79.0%, respectively).

**Figure 3.**
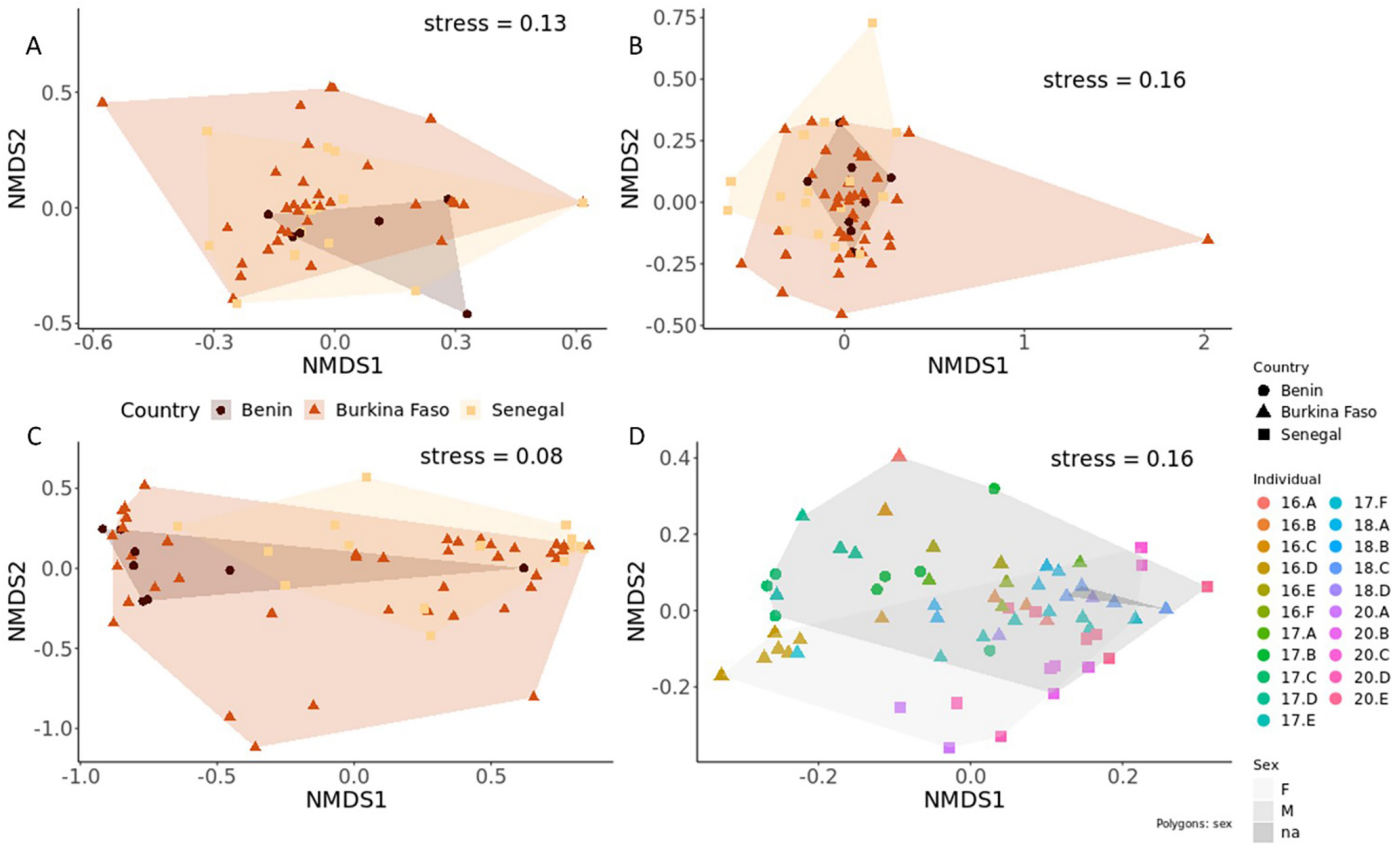
*n*MDS plots based on Bray–Curtis dissimilarities (*K* = 3 dimensions) for the (A) haemoparasite, (B) microbiome and (C) nemabiome communities, with hulls coloured by country and samples as points. (D) Total parasite communities, with points representing samples and coloured to show individual lions, with hulls shaded to show sex and point shape to show country.

For kinetoplastids, we found a greater prevalence (19/21 individuals) coupled with higher diversity and variation among individuals compared with apicomplexans. A total of 606 kinetoplastid ASVs were identified with richness per individual ranging from 2 to 323 (individual 20.C, a female from Senegal; table 1). The overall mean ASV richness was 67.5, and varied by population (Benin = 37.5; Burkina Faso = 49.6; Senegal = 103) but was not statistically different (repeated measures ANOVA: (*F*_(2, 16)_ = 1.571, *p* = 0.238, *η*² = 0.11). Like apicomplexans, there was no statistical difference between the mean richness and sex of individuals (repeated measures ANOVA: (*F*_(2, 16)_ = 0, *p* = 0.98). The majority of kinetoplastid detections were from the family Trypanosomatida (electronic supplementary material, figure S1) and included *Herpetomonas* spp., a genus of insect parasites associated with flies [65]. The other families detected were Neobodonida in six lion individuals and Parabodonida with *Parabodo caudatus* detected in 14 lion individuals (figure 2). In Benin, *Parabodo caudatus* made up 98.4% of sequences. In Burkina Faso, *Herpetomonas* made up 69.9%, while the *Trypanosomatidae* sp. ‘strain_Eva’ made up 48.2% of sequences. When consolidating both apicomplexan and kinetoplastid detections, the overall haemoparasite communities differed significantly among individuals (R ANOSIM: 0.41 , *p* < 0.001) as well as among populations (R ANOSIM: 0.18, *p* = 0.001, figure 3).

### 3.2. Nemabiome diversity

**Figure 4.**
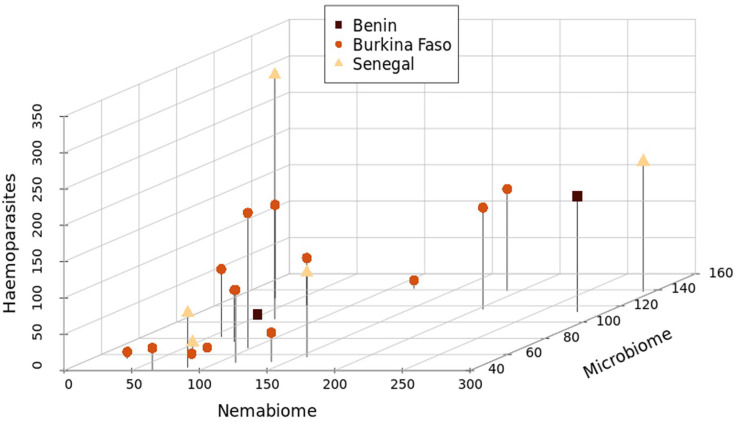
Three-dimensional scatter plot depicting the amplicon sequence variant (ASV) richness of haemoparasite, nemabiome and microbiome diversity in the individual lions.

We detected nemabiome sequences in all lion individuals. After the removal of singletons and doubletons, we identified 2311 unique nematode ASVs across sampled lion populations. Nemabiome ASV richness per individual lion ranged from 26 to 292 (table 1). The mean ASV richness varied among populations: Benin = 44.6, Burkina Faso = 41.2, Senegal = 65.8; however, these differences were not statistically significant (repeated measures ANOVA: *F*_(2,18)_ = 2.409 , *p* = 0.118). In contrast, sex was significantly associated with observed ASV richness. Male lions had significantly higher ASV richness compared with females across populations (repeated measures ANOVA: *F*_(1,18)_ = 6.127, *p* = 0.02, *η*² = 0.11). The nemabiome communities differed significantly among individuals (R ANOSIM: 0.16, *p* = 0.02), as well as among populations (R ANOSIM: 0.10, *p* = 0.02, figure 4). The majority of detections, for each country, were from the order Rhabditida that were found in 64/65 samples, an order of microbivorous nematodes that includes species known to be soil dwelling or zooparasitic (figure 2). These made up 94.5% (Benin), 81.3% (Burkina Faso) and 58.3% (Senegal) of sequencing read counts. The next most abundant detections were from the genus *Ancylostoma*, which were found in 17/21 individuals (figure 3). *A. duodenale*, a hookworm known to be found in the small intestine of humans, cats and dogs, was detected in 16 individuals and *A. tubaeforme*, one of the most common hookworms to infect cats, was detected in 17 individuals. Strongylida, an important gastrointestinal lungworm was detected in 17/21 individuals, while the feline lungworm *Oslerus rostratus* was detected in 8/21 individuals (figure 3). Sequences most closely related to *Gurltia paralysans*, a poorly studied presumed South American species of feline Metastrongyloid, were detected in 8/21 individuals. *Chromodorea* was most prominent in individuals from Burkina Faso (electronic supplementary material, figure S1). Additionally, a concomitant ‘false positive’ may include *Caenorhabditis plicata*, a species phoretic on carrion beetles, detected in one sample (for individual 16.C).

### 3.3. 16S-based bacteria community profiling (i.e. microbiome)

Microbial sequences were detected in all lion individuals, and after the removal of singletons and doubletons, we detected 2240 microbiome ASVs across populations. The observed ASV richness per individual ranged from 41 to 142 (mean = 86.7; table 1) and varied by country: Benin = 110, Burkina Faso = 85.2, Senegal = 81.8. However, microbiome ASV richness was not statistically different among populations (repeated measures ANOVA: *F*_(2,18)_ = 0.61, *p* = 0.55) or sexes (repeated measures ANOVA: *F*_(2,18)_ = 0.44, *p* = 0.51). Compared with the ASV richness, the composition of microbiome communities was significantly different among individuals (R ANOSIM: 0.52, *p* < 0.001) as well as between sexes (R ANOSIM: 0.24, *p* < 0.001) and populations (R ANOSIM: 0.19, *p* = 0.009; figure 4). SIMPER analysis revealed the detections of non-specific bacteria, *Collinsella*, *Clostridium*_XI and Lachnospiraceae cumulatively contributed to 73.8% dissimilarity between males and females. These species also contributed to the dissimilarity among populations. When comparing Benin and Burkina Faso (electronic supplementary material, figure S1), *Virgibacillus* was only detected in samples from Benin, and significantly contributed to dissimilarity (*p* = 0.01). The primers amplified predominantly bacterial ASVs (*n* = 2182) with few from phyla Archaea (*n* = 58). In each population, the majority of detections comprised of non-specific bacterial sequences, making up 51.9% (Benin), 71.0% (Burkina Faso) and 94.2% (Senegal) of sequencing reads. Of the remaining sequences, 12 phyla were detected with Actinobacteria and Firmicutes making up the major proportion of diversity in each individual (figure 2). Twenty classes were detected, with Actinobacteria, Clostridia and Bacilli making up the most abundant detections (following the non-specific bacterial sequences) in each population (figure 2). In Benin and Burkina Faso, Actinobacteria made up 25.6% and 16.0% of sequencing reads, respectively, while Clostridia were the next most abundant detection (2.5%).

### 3.4. Parasite co-occurrence

When all parasite groups were analysed together, the composition of each individual lion’s parasite community significantly differed (R ANOSIM: 0.51, *p* < 0.001; figure 3). Differences between parasite communities for each sex were less pronounced, but still statistically significant (R ANOSIM: 0.10, *p* = 0.03), and there was no statistical difference between the parasite communities among populations (R ANOSIM: 0.08, *p* = 0.08). There were significant correlations between nemabiome ASV richness and apicomplexan ASV richness (Spearman’s *R* = 0.46, *p* = 0.04), and nemabiome ASV richness and haemoparasite (the sum of apicomplexan and kinetoplastid) ASV richness (Spearman’s *R* = 0.50, *p* = 0.02; figure 4). We also found significant interactions between sex and population with the ASV richness of some of the parasitic groups and the microbiome. For example, sex and microbiome richness interacted to significantly affect kinetoplastid richness. Additionally, we found that population interacted with microbiome richness to significantly affect both nemabiome and haemoparasite richness (the sum of apicomplexan and kinetoplastid richness) among individuals.

## 4. Discussion

Lions exemplify the complex feedbacks between natural and human systems through their consumptive and non-consumptive effects on structuring prey communities, while remaining of great conservation concern with cultural and economic significance [66,67]. The critically endangered West African lion population now occupies only 1.1% of its historical range—Senegal, Nigeria, Burkina Faso, Niger and Benin—due to habitat loss, prey depletion and retaliatory killing in response to livestock depredation [35]. Across their remaining range, African lions are almost exclusively confined to protected areas that are experiencing immense human pressures (e.g. [50,68]). Sympatry with humans and domestic animals does increase exposure to trophically transmitted helminths and infectious diseases that cause mortality, morbidity and alter the behaviour for wildlife, including lions [69,70]. Pressures on African lion populations will intensify in the future, as the demand for livestock products is expected to double by 2050 [71,72]. Thus, understanding the composition and variation of parasite communities provides insight into lions’ exposure to infection, their ability to cope with parasite burdens and how these pressures may compromise population resilience. Such knowledge also reveals how changes in lion abundance could reshape parasite transmission networks, with potential cascading effects on co-occurring wildlife, livestock and broader ecosystem function [73,74].

The consequences of species rarity on parasite communities can manifest as homogenization within host individuals that alter transmission dynamics throughout the ecosystem [75,76]. Common hosts with large populations, are probabilistically more likely to have varied diets and habitat use patterns, that influence their exposure rates to parasites and extinction risks [77,78]. As populations shrink in size, the variation across ecological and evolutionary characteristics that existed across individuals within the species also declines. As such, smaller populations experience increased vulnerability to the ‘extinction vortex’ and extirpation risks from single catastrophic events [79]. We found that the Senegal population exhibited lower nemabiome richness, consistent with our hypothesis and previous research showing that smaller host populations often support fewer parasites [36]. In contrast, microbiome diversity did not differ significantly between populations, which may reflect either the limitations of our sample size, the environmental homogeneity within protected areas or comparable diets across populations due to overlapping prey availability. African lions may also simply have a ‘core’ microbiota despite geographical separation [80], explaining why isolation reduced their nemabiome diversity and not their microbiome. Coextinction further illustrates how reduced host population sizes may result in fewer parasite species, as smaller populations often lose both host-specific parasites and the symbiotic relationships that sustain them [81].

Inventories of parasite variation among populations help provide assessments for predicting effects of land-use change on the emergence and distribution of disease burden particularly between the domestic-wildlife interface [82]. Previous studies in carnivores have shown how parasite communities may be influenced; three tiger (*Panthera tigris*) populations in Nepal exhibited distinct gut microbiota corresponding to variation in genetic diversity and anthropogenic pressures [83]. In a comparative study across caracal (*Caracal caracal*) populations in South Africa, individuals in the urban edge population harboured more *Anaplasma* species, tick-borne pathogens and other parasites across human-modified environments [84]. Other systems also highlight spillover concerns between domestic species and threatened carnivores due to increasing anthropogenic pressures (e.g. red pandas *Ailurus fulgens* in Nepal [85]; red wolves *Canis rufus* in the USA [86]). With prevailing global environmental and land-use changes, large carnivores are shifting and contracting ranges that alters community assemblages [30]. Resultant variation in species sympatry will also induce differences in associated parasite assemblages across populations. For example, spatial overlap of grey wolves (*Canis lupus*) with a highly dense cougar (*Puma concolor*) population correlated with higher seropositivity of *Toxoplasma gondii* in Yellowstone National Park, which had direct impacts on risk-tasking behaviours [87]. Ultimately, understanding the distribution and assemblage of variation within parasite communities across scales from individuals to populations to species is a necessary precursor to anticipate global disease patterns and the potential emergence of future pandemics. This is particularly important for ‘interface’ species that reside in close proximity to humans or areas with higher encounters with domestic species. However, we recognize the presence of these parasites does not automatically indicate disease or a spillover event without subsequent health studies.

Given interactions between parasite exposure and consumptive patterns, the observed variation in parasite affiliates among lion individuals particularly in the nemabiome and microbiome may reflect differences in preferred prey or at minimum recent diet selection. For example, the alpha diversity of gut microbes decreased with prey densities for individual spotted hyenas (*Crocuta crocuta*) in the Masai Mara in Northern Kenya reflecting seasonal variation in hunting strategies and scavenging behaviour [88]. Similarly, the diet of grey wolves varied with association to the presence of helminths in Manitoba, Canada in that individuals that relied more on beaver instead of white-tailed deer had fewer cestodes [89]. These patterns support that prey selection plays a vital role in shaping parasite communities in carnivores, given the strong diet-gut microbe interdependence [90]. As such, future enquiry into relationships between diet and parasite exposure are needed in Africa lions to discern how these interactions contribute to inducing variation among individuals to affect their behaviour, ecology and demography.

## 5. Limitations

There remain some limitations to the molecular detection of parasites and considerations regarding the inference of results. Metabarcoding relies on the ability to successfully extract and amplify good-quality DNA. DNA from faecal samples is typically degraded due to environmental exposure and microbial activity [91–93]. Additionally, the concentration of parasite DNA in faeces is often much lower than that of host DNA, raising the likelihood of false negative results [94]. Further work is needed to distinguish between parasite DNA and other sources, such as ingested material (e.g. prey species) or environmental contamination. Collecting faecal samples deposited at various times is particularly subject to environmental contamination due to wind, rain, soil and even insect exposure that alter interpretations of our prevalence and parasite occurrence data. For example, DNA from organisms like detritivores or arthropod parasites may lead to non-target detections, such as our detection of *Herpetomonas* spp*.*, a genus associated with insect parasites [65]. Additionally, faecal samples may not reliably reflect the true infection status due to the inherent complexity of parasite life cycles [95], as samples may only contain traces of the parasite at a particular stage or may entirely miss stages that occur within other hosts or outside the gastrointestinal tract of the sampled host [96]. The effectiveness of metabarcoding is heavily dependent on the quality and completeness of reference databases to distinguish between closely related species or strains and reduce sampling bias [97]. Finally, our study does not provide the complete picture of the parasite assemblage that plagues lions, given the exclusion of viruses and ectoparasites and limited taxonomic resolution for bacteria.

## 6. Conclusion

The parasite diversity identified in this study has important conservation implications for critically endangered populations of West African lions. Even with a small sample size of 20 individuals, new insights emerged about health challenges that could be further exacerbated under future climate and land-use changes. We highlight parasite surveillance in wild lions as a tool for understanding pathogen ecology and their potential sentinel roles in changing landscapes. Our detections of parasites like *Sarcocystis* and *Ancylostoma*, known to cause morbidity in carnivores, pose both particular risks and management opportunities that warrant further investigation. The detection of zoonotic parasites, such as *Ancylostoma duodenale*, raises concerns about pathogen transmission between wildlife, livestock and humans, highlighting the need to monitor and mitigate parasite exposure, especially in areas where human–wildlife interactions are increasing [98,99]. Conservation and management require an understanding of interactions between organisms, including predator–prey, and host–parasite interactions with particular caution needed to discern pathogenicity [24]. Faecal samples can contain abundant information on diet, genetics and parasites and offer a non-invasive way to collect data from threatened or endangered wild vertebrates [100]. This approach offers a promising pathway for monitoring biodiversity, evaluating ecosystem health and implementing effective conservation strategies, even in challenging conditions where traditional methods may be less feasible or effective. Our study leverages advanced molecular techniques, providing insights into the health and ecosystem dynamics affecting a species of great cultural, economic and ecological value. By integrating metabarcoding with microsatellite analysis, our research sheds light on parasite load of these apex predators at the individual and population scale as well as demonstrates the utility of molecular tools in glean information for wildlife conservation [101].

## Data Availability

Data are available in the electronic supplementary material [102].
